# A bioactive compound isolated from Duku ( *Lansium domesticum* Corr) fruit peels exhibits cytotoxicity against T47D cell line

**DOI:** 10.12688/f1000research.21072.2

**Published:** 2021-04-23

**Authors:** Khusnul Fadhilah, Subagus Wahyuono, Puji Astuti

**Affiliations:** 1 Faculty of Pharmacy, Universitas Gadjah Mada, Yogyakarta, 55281, Indonesia; 2 Departement of Biology, Faculty of Pharmacy, Universitas Gadjah Mada, Yogyaka, 55281, Indonesia

**Keywords:** Lansium domesticum Corr., cytotoxic, T47D cell line, Lamesticumin A

## Abstract

**Background:** Breast cancer is a major health problem for women globally. Many attempts have been promoted to cure cancer by finding new anticancer medicines from natural resources. Despite the richness of biodiversity discovered, there are some natural resources that remain unexplored. Fruit peels of Duku ( *Lansium domesticum* Corr.) are rich with compounds that may have the potential to be developed as anticancer drugs. This study aimed to isolate cytotoxic compounds from the fruit peels of *L. domesticum *and assess their cytotoxic nature against T47D cells.

**Methods:** Powdered peels were macerated with ethyl acetate and the filtrate was evaporated to give EtOAc extract A. Dried extract A was triturated with n-hexane to give n-hexane soluble fraction B and insoluble fraction C. The cytotoxic nature of these three  samples were assessed using MTT assay using T47D cells and doxorubicin as a control.

**Results:** Fraction C that showed the smallest IC50 (25.56 ± 0.64μg/mL) value compared to  extract A and fraction B. Fraction C was further fractionated by vacuum liquid chromatography to give 6 subfractions. Subfraction 2 showed a single compound based on thin layer chromatography, and this compound was identified as Lamesticumin A on the basis of its spectroscopic data. Lamesticumin A demonstrated cytotoxic activity against T47D cell lines with an IC _50_ value of 15.68 ± 0.30µg/mL.

**Conclusions:** Further research is needed to investigate the potential of the natural compound Lamesticumin A derived from *L. domesticum* fruit peel as an anticancer therapy.

## Introduction

The most frequent cancer in women and that which causes the highest mortality is breast cancer. In Indonesia, it was reported that approximately 21% of cancer deaths among women were due to breast cancer ^1^. Therefore, new medicines to eradicate this type of cancer is required. Duku ( *Lansium domesticum* Correa) widely grows in Indonesia. Traditionally, *L. domesticum* bark and seeds have been used to treat dysentery and fever ^2^. Based on previous studies, chloroform and methanol extracts of *L. domesticum* displayed cytotoxic activity on murine melanoma (B _16_F _10_) and colon cancer (HT29) cells ^3^. In addition, it has been shown that ethanol and ethyl acetate fractions of the peel have a deterrent activity on DNA damage in lymphoblast cells induced by H _2_O _2_ exposure ^4^. Onoceranoid-type of triterpenoids have been isolated from twigs and leaves of *L. domesticum*, and these compounds showed antibacterial and antimutagenic activities ^5, 6^. In this study, the cytotoxic effects of compound extracted from the peels of *L. domesticum* are assayed against breast cancer T47D cells.

## Methods

### Plant material

The fruits of *L. domesticum* were collected on March 2018 from Bantul, Yogyakarta (GPS : -7.871098, 110.394854) and identified at the Department of Pharmaceutical Biology, Faculty of Pharmacy, Universitas Gadjah Mada.

### Chemicals and equipment

Organic solvents (methanol, ethyl acetate, chloroform, n-hexane) used were pro analytical grades obtained from Merck. Silica gel F _254_, Silica gel PF _254_, (Merck), RPMI 1640, Fetal Bovine Serum, Penicillin-Streptomycin, Fungizon, Sodium bicarbonate (Gibco), HEPES (Invitrogen), Phosphate Buffered Saline, MTT (Sigma Aldrich cat. M5655), Doxorubicin (Sigma Aldrich). Infrared (KBr) spectrum was obtained from spectrophotometer (Shimadzu) using a method previously described by Ashokkumar and Ramaswamy ^7^. Ultraviolet spectrum (CHCl _3_) was obtained from UV spectrophotometer (Hitachi UH 5300). Sample (1 mg) were diluted in 1 mL CHCl _3_ and was run between 200–400 nm. Spectra of ^1^H- and ^13^C- NMR in CDCl _3_ solvent were measured using JEOL JNM-ECZ 500R/S1 at 500 MHz.

### Extraction and fractionation

The peel was separated from the fruit, dried in oven 50°C for 24 hours and powdered using a blender. Powdered *L. domesticum* fruit peel (200g) was macerated with ethyl acetate (EtOAc; 2 L) overnight. This solution was filtrated (0.45μm) and the filtrate was evaporated to dryness with a rotary evaporator set at 50°C, to give dried EtOAc extract (A, 50.13 g). In order to separate the extract into non-polar and polar fractions, 6 g of extract A was diluted 5 times using n-hexane (20 mL) to give soluble n-hexane fraction B (supernatant; 3.03 g) and insoluble n-hexane fraction C (residue; 2.83 g).

**Figure 1.**
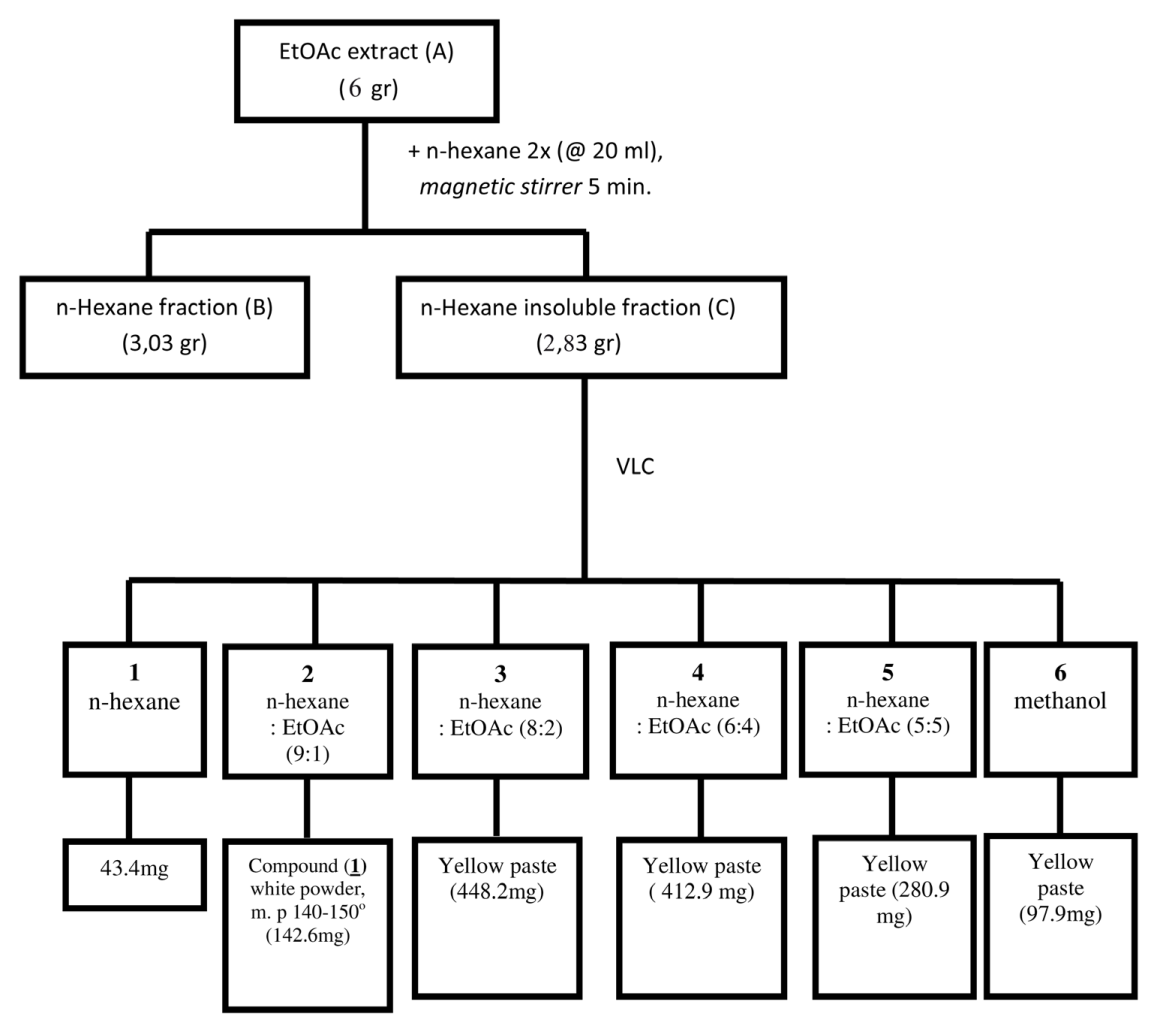
Isolation process of cytotoxic compound 1 (subfraction 2) from *Lansium domesticum* fruit peels.

Fraction C was the most active among other fractions (see *Results*), and was therefore further fractionated using vacuum liquid chromatography as described by Mae Sri Hartati *et al*. ^8^. In brief, using silica gel preparation grade (15g) as stationary phase this was eluted with n-hexane and increasing amounts of ethyl acetate. Six subfractions were obtained and subfraction 2 contained a major compound which appeared as white crystals (referred to as compound 1). Compound 1 was obtained as a single compound from subfraction 2, while the other subfractions still contained various compounds. Compound 1 (142.6 mg), had a melting point at 140–150°C ( Figure 1). Compound 1 was identified using spectroscopy data such as ultraviolet (UV), infrared (IR), ^13^C-NMR and ^1^H-NMR (see section *Chemicals and equipment*).

### Cytotoxic assay

The bioassay followed the methodology described by Bahuguna *et al*. ^9^ with modifications. In brief, 100 μl T47D and Vero cell line (in RPMI mediaFaculty of Medicine, Universitas Gadjah Mada) were placed in each well of a 96 well micr oplates, resulting in 1 × 10 ^4^ cells/well. The cells were incubated for 24 hours at 37°C in a CO _2_ incubator.

Extract A, fractions B and C and compound 1 (5mg) were dissolved in DMSO (50 μL). Serial concentrations of extract and fractions (50, 25, 12.5, 6.25, 3.125 μg/mL), compound 1 (25, 12.5, 6.25, 3.125 μg/mL) and doxorubicin (positive control; 0.5, 0.25, 0.125, 0.0625, 0.0312 μg/mL) were obtained. Cells were treated with the dose dependent samples and incubated for 24 hours at 37°C. The culture medium was removed by pipette, and MTT solution (100μL) was added to each well and incubated for 4 hours at 37°C. After incubation, stop solution (10% SDS, 100 μL) was added to each well and let stand at room temperature for 24 hours.

Absorbance was measured by microplate reader (Bio Rad) at 595 nm. positive control The data generated were used to plot a dose-response curve and IC _50_ of the samples was determined.

### Statistical analysis

The IC _50_ values were analyzed by one-way ANOVA with statistical significance P < 0.05 using IBM SPSS ver.23.

## Results

### Compound 1 characterization

Identified as Lamesticumin A.

White crystal. IR (KBr) *v _max_* cm ^-1^: 3074, 2960, 1712; UV (MeOH) *λ _max_* 236,5; ^1^H, ^13^C-NMR: see Table 1; m/z 502; (Calculated for C _31_H _50_O _5_)

**Table 1.** 13C-NMR spectrum (500 MHz, CDCl3) of compound 1, Lamesticumin A.

Position	^1^H-NMR (J, Hz)		^13^C-NMR
	δ (ppm)	Multiplicity	δ (ppm)
**1**	1.2	2H, triplet (7.0)	27.98
**2**	2.1	2H, triplet (6.8)	29.18
**3**	-	-	147.60
**4**	-	-	51.78
**5**	0.9	1H, triplet (7.1)	50.78
**6**	1.4	2H, multiplet	27.51
**7**	1.9	2H, triplet (6.8)	28.75
**8**	-	-	122.10
**9**	1.1	1H, triplet (7.0)	48.91
**10**	-	-	47.68
**11**	1.4	2H, multiplet	30.59
**12**	1.7	2H, multiplet	29.67
**13**	1.13	1H, triplet (7.0)	41.71
**14**	-	-	135.91
**15**	5.4	1H, triplet (6.8)	113.93
**16**	1.7	2H, dd (7.0, 6.8)	30.59
**17**	1.1	1H, triplet	31.81
**18**	-	-	38.85
**19**	1.2	2H, multiplet	32.09
**20**	2.1	2H, multiplet	33.15
**21**	-	-	148.10
**22**	-	-	147.20
**23**	1.7	3H, singlet	23.37
**24**	1.9	3H, singlet	23.76
**25**	0.8	3H, singlet	16.16
**26**	4.8	2H, dublet (9.1)	107.4
**27**	1.7	3H, Singlet	17.91
**28**	0.7	3H, singlet	14.37
**29**	4.6	2H, dublet (9.2)	114.23
**30**	1.6	3H, singlet	22.88

^1^H- and ^13^C- NMR (CDCl _3_) spectra were obtained from JEOL JNM-ECZ 500R/S1, 500 MHz

**Figure 2.**
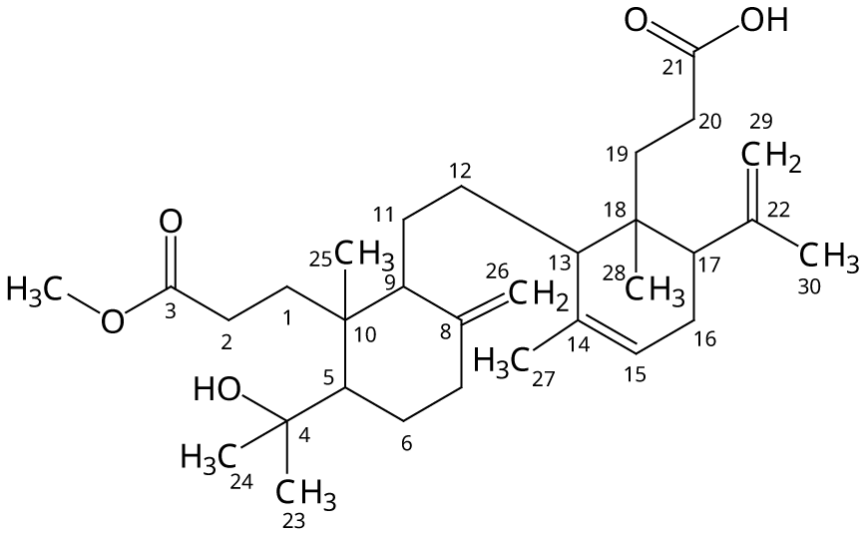
Isolated compound 1, Lamesticumin A.

The infrared spectroscopy (KBr) spectrum of 1 showed a broad band at 3400–2800 cm ^-1^, which indicated the presence of –OH group, specifically –COOH due to intermolecular bonding. This data is supported by the appearance of –C=O at 1712 cm ^-1^. Compound 1 displayed UV absorption at 236,5 nm. The ^13^C-NMR spectrum (500 MHz, CDCl _3_) of compound 1 showed 30 carbons ( Table 1). There were two down field carbon signals (δ, 147.6 and 148.1 ppm) identified as C=O signal carbons. Two characteristic terminals =CH _2_ signals (δ, 107.4 and 114.2 ppm) were observed, and this identity was confirmed by 2D (Het-Cor) NMR technique. Based on ^13^C-NMR and ^1^H-NMR data, compound 1 ( Figure 2) was identified as Lamesticumin A (C _31_H _50_O _5_, m/z, 502) which was previously isolated from *L. domesticum* twigs ^5^.

### Cytotoxicity of extract A, fractions B and C, and compound 1

**Figure 3.**
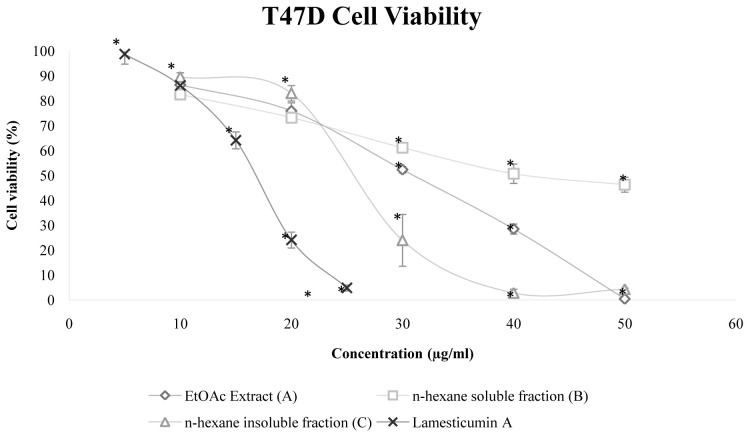
T47D breast cancer cell line viability after treatments with extracts from *Lansium domesticum* fruit peels. Error bar shows standar deviation (SD, n=3). symbol (*) indicates statistical significance (p<0.05) Error bars shows standard deviation.

The cytotoxicity of extract A, fractions B and C, compound 1 and doxorubicin (positive control) is shown in Table 2. Fraction C was the most cytotoxic (IC _50_ 25.57 μg/mL) compared with extract A (29.41 μg/mL) and fraction B (43.51 μg/mL). The IC _50_ of the isolated compound from fraction C, compound 1/Lamesticumin A was 15.68 μg/mL. All samples inhibited T47D cell growth in a dose dependent behavior ( Figure 3).

**Table 2.** IC _50_ values of extract, fractions and isolated compound 1 against T47D breast cancer cell line and normal cell line (Vero).

Sample	IC _50_ (μg/ml), mean ± SD	
	T47D	Vero
**Extract A**	29.41 + 0.67	66.10 ± 3.26
**Fraction B**	43.51 ± 1.77	77.53 ± 5.03
**Fraction C**	25.56 ± 0.64	81.12 ± 1.12
**Lamesticumin A**	15.68 ± 0.30	47.46 ± 2.63
**Doxorubicin**	0.18 ± 0.01	11.02 ± 3.63

## Discussion

In this study, the cytotoxic activity of Lamesticumin A, derived from the peel of *L. domesticum,* was demonstrated in the T47D cell line with IC _50_ 15.68 (μg/ml). The T47D cell line is an epithelial breast cancer cell subtype luminal A cell line that express estrogen and progesterone receptors ^10^. Based on National Cancer Institute guidelines, a natural compound has potent anticancer activity if it has IC _50_ <4 μg/ml or 10 μM ^11^.

Many triterpenoid compounds have been previously isolated from *L. domesticum*. Most of these compounds are UV inactive or have no strong UV absorbance because triterpenoid’s lack of a conjugated functional group ^12^. Lansiosida A and Dukunolida A has been isolated from n-hexane extract of *L. domesticum* fruit peel ^13, 14^. Lamesticumin A is an onoceranoid-type triterpenoid, isolated previously from *L. domesticum* twigs, that has antibacterial activity against *Staphylococcus aureus, Staphylococcus epidermidis, Micrococcus luteus, Bacillus subtilis, Micrococcus pyogenes* and *Bacillus cereus* with minimum inhibitory concentration of <15 μg/ml ^5^. Another onoceranoid-type triterpenoid Lansium acid I-IX were isolated from *L. domesticum* leaves, which was reported to have antimutagenic activity ^6^.

Based on several *in vitro* tests, some terpenoid compounds had anticancer activity. Sesquiterpene lactone compounds are known to inhibit Nf-kB, thereby inducing apoptosis ^15^. Celastrol has anticancer properties by regulating various transcription factors, angiogenesis processes, cell cycle arrest and induction of apoptosis ^16^. Betulinic acid can induce apoptosis in HT-29 colon cancer cells and acts as a chemosensitizer for chemotherapeutic agents in wildtype adenocarcinoma cancer cells (SNU-C5/WT) ^17^. Clematangoticosides D and F from *Clematis tangutica* are known to have cytotoxic activity against human gastric cancer cell line (SGC-7901) with IC _50_ 24.22 and 21.35 μM, respectively ^18^. Cycloartane-type and oleanane-type triterpenoids from *Ligularia przewalskii* show cytotoxicity in Hela, HEPG2, SGC7901, MDA-MB-231, HL-60, and Lewis cell lines with IC _50_ 8.40–24.39 μM ^19^.

It has been reported that natural compounds combined with low doses of antineoplastics can increase effectiveness and reduce toxic effects ^20^. Betulinic acid can induce apoptosis when combined with 5-fluorouracil, irinotecan and oxaliplatin ^4^. Ursolic acid (UA), a pentacyclic triterpenoid, is known to have anticancer activity through interfering with multiple signaling pathways. Furthermore, UA has been shown to act as a chemosensitizing agent to increase the effect of conventional anticancer drugs ^21^, and to increase the effect of doxorubicin by increasing the cellular amount of the drug in the MCF-7 cell line ^22^. Further study is needed to investigate the possibility of Lamesticumin A to be combined with doxorubicin for its potential to have synergistic effect.

## Conclusions

Extract, fractions and Lamesticumin A derived from the peel of *L. domesticum* showed cytotoxic activity against the T47D breast cancer cell line. Further research is needed to investigate the potential of the natural compound Lamesticumin A derived from *L. domesticum* fruit peel as an anticancer therapy.

## Data availability

### Underlying data

Zenodo: A bioactive compound isolated from Duku (Lansium domesticum Corr) fruit peels exhibits cytotoxicity against T47D cell line, http://doi.org/10.5281/zenodo.3539670 ^23^.

This project contains the following underlying data:

- UV, infrared, ^13^C-NMR and ^1^H-NMR spectra of compound 1.
- Cell viability and IC _50_ values of extract A, fractions B and C, compound 1 and doxorubicin in T47D cell line.

Data are available under the terms of the Creative Commons Attribution 4.0 International license (CC-BY 4.0).
