# Supplementary material for: A bioactive compound isolated from Duku ( Lansium domesticum Corr) fruit peels exhibits cytotoxicity against T47D cell line
Source: F1000Res. 2021 Apr 23;9:3. Originally published 2020 Jan 6. [Version 2] doi: 10.12688/f1000research.21072.2 (PMC8185580; doi:10.12688/f1000research.21072.2)
Supplement: Peer review history

# Peer review: Reviewer response for version 2

**DOI:** 10.5256/f1000research.52564.r83806

**Published:** 2021-06-07

**Authors:** Ratana Banjerdpongchai

**Affiliations:** 1 Department of Biochemistry, Chiang Mai University, Chiang Mai, Thailand

The manuscript is improved according to the comments of the reviewer’ major comments. However, the incidence of breast cancer in female should be updated to be in year 2000 of GLOBOCAN.

 For minor points: The scientific names of plants should be all in italic. Sometimes the authors used g and sometimes as gr, it should be consistent with each other throughout the manuscript, including the diagram of extraction in Figure 1: Isolation process of cytotoxic compound 1 (subfraction 2) from *Lansium domesticum* fruit peels. There are still typo and grammar errors, which have been highlighted here. For examples, ml vs mL. et al sometimes in italic and sometimes was not. In References, there is an et al. not in italic form. The usage of IC50, the number should be in subscript form.

 After correction of the points mentioned above, the manuscript can be indexed under the publisher’s supervision and handling.

Is the work clearly and accurately presented and does it cite the current literature?

Yes

If applicable, is the statistical analysis and its interpretation appropriate?

Partly

Are all the source data underlying the results available to ensure full reproducibility?

Yes

Is the study design appropriate and is the work technically sound?

Yes

Are the conclusions drawn adequately supported by the results?

Yes

Are sufficient details of methods and analysis provided to allow replication by others?

Yes

Reviewer Expertise:

Programmed cell death, Natural products, Antioxidants, Cancer

I confirm that I have read this submission and believe that I have an appropriate level of expertise to confirm that it is of an acceptable scientific standard.

# Peer review: Reviewer response for version 1

**DOI:** 10.5256/f1000research.23191.r64744

**Published:** 2020-09-17

**Authors:** Deden Derajat Matra

**Affiliations:** 1 Department of Agronomy, Bogor Agricultural University, Bogor, Indonesia

The author did not describe the maturity degree from samples because the different stages of maturity may change secondary metabolites that concern effect to the bioactive compound.

 How to handling samples after harvesting is important too. Please, author, describe more? 

 In conclusion, what kind of peel fresh or processed is best consumed as the higher potential to anticancer therapy.

Is the work clearly and accurately presented and does it cite the current literature?

Yes

If applicable, is the statistical analysis and its interpretation appropriate?

Partly

Are all the source data underlying the results available to ensure full reproducibility?

Yes

Is the study design appropriate and is the work technically sound?

Yes

Are the conclusions drawn adequately supported by the results?

Partly

Are sufficient details of methods and analysis provided to allow replication by others?

No

Reviewer Expertise:

Agricultural Plant Science, Horticulture, AgronomyPhysiology, Bioinformatics, Molecular Biology.

I confirm that I have read this submission and believe that I have an appropriate level of expertise to confirm that it is of an acceptable scientific standard.

# Peer review: Reviewer response for version 1

**DOI:** 10.5256/f1000research.23191.r70005

**Published:** 2020-08-26

**Authors:** Ratana Banjerdpongchai

**Affiliations:** 1 Department of Biochemistry, Chiang Mai University, Chiang Mai, Thailand

Major points:

 The extracts and purified bioactive compound, Lamesticumin A, from this plant, is not novel, since it was isolated from the twigs from this same herb in the previous report. The anticancer activity of the active compound is not promising since it is more than 4 microgram/mL or 10 micromolar, the level of which the National Cancer Institution defines is 4 microgram/mL or less than this. However, the authors discussed of using this compound as a chemosensitizing agent, which various steps still are needed before launching it as a therapeutic anti-cancer drug. The authors should explore the cytotoxic effects of the extracts, EtOAc extract (A), N-hexane fraction (B), N-hexane insoluble fraction (C) and compound 1 (Lamesticumin A) on normal cells.

 In Figure 3, the percent cell viabilities of EtOAc extract (A), N-hexane fraction (B), N-hexane insoluble fraction (C) and compound 1 (Lamesticumin A) should be presented with the statistical significance by using asterisks, such as *p<0.05, **p<0.01, and so on, above the dots.

 Minor points:

 The sources of chemicals and instruments should indicate the city and country that they are produced from. Sometimes the authors used gr and sometimes g, it should be consistent with each other. In subtopic “Cytotoxic assay”, in 3 ^rd^ – 4 ^th^ lines, the authors should rewrite the sentences. There are many grammar and various typological errors, such as the number of g it should be 43.4 rather than 43,4, etc. For the previous findings, the sentences should be in present tense, but for the authors’ research data in the Results and Discussion parts, the sentences would be in past tense. For MDA231, it should be MDA-MB-231 breast cancer cells. For MCF, it should be clarified as MCF-7 or not? In Ref. No. 12, there is no information of company, city, country of publication, is it a book? Ref. No. 21, the name of journal is full name, whereas others are in abbreviated forms. It should be consistent with each other.

Is the work clearly and accurately presented and does it cite the current literature?

Yes

If applicable, is the statistical analysis and its interpretation appropriate?

Partly

Are all the source data underlying the results available to ensure full reproducibility?

Yes

Is the study design appropriate and is the work technically sound?

Yes

Are the conclusions drawn adequately supported by the results?

Yes

Are sufficient details of methods and analysis provided to allow replication by others?

Yes

Reviewer Expertise:

Programmed cell death, Natural products, Antioxidants, Cancer.

I confirm that I have read this submission and believe that I have an appropriate level of expertise to confirm that it is of an acceptable scientific standard, however I have significant reservations, as outlined above.

## References

[1] : Anticancer activities of the extract from Longkong (Lansium domesticum) young fruits. *Pharm Biol* .2012;50(11) : 10.3109/13880209.2012.682116 1397-407 10.3109/13880209.2012.682116 22849519

# Response

**Published:** 2021-01-11

**Authors:** Khusnul Fadhilah

**Affiliations:** Universitas Gadjah Mada, Indonesia

In the revised version, we present cytotoxic data on normal cells (Vero cell line) in Table 2 and Figure 3 has been revised as recommended by the reviewer for major point revision. Besides, we also present the minor points revision such as grammar and typological errors corrections.
